# IL-1 signal affects both protection and pathogenesis of virus-induced chronic CNS demyelinating disease

**DOI:** 10.1186/1742-2094-9-217

**Published:** 2012-09-17

**Authors:** Byung S Kim, Young-Hee Jin, Liping Meng, Wanqiu Hou, Hyun Seok Kang, Hey Suk Park, Chang-Sung Koh

**Affiliations:** 1 Department of Microbiology-Immunology, Northwestern University Medical School, 303 East Chicago Ave, Chicago, IL, 60611, USA; 2 Biomedical Laboratory Sciences, Graduate School of Medicine, Shinshu University, Matsumoto, Nagano, 390-8621, Japan; 3 Visiting Assistant Researcher at UCLA, Los Angeles, CA, USA

**Keywords:** IL-1R, TMEV, Demyelination, CNS, T cell responses, IL-1, IL-1R KO mice, Th17

## Abstract

**Background:**

Theiler’s virus infection induces chronic demyelinating disease in mice and has been investigated as an infectious model for multiple sclerosis (MS). IL-1 plays an important role in the pathogenesis of both the autoimmune disease model (EAE) and this viral model for MS. However, IL-1 is known to play an important protective role against certain viral infections. Therefore, it is unclear whether IL-1-mediated signaling plays a protective or pathogenic role in the development of TMEV-induced demyelinating disease.

**Methods:**

Female C57BL/6 mice and B6.129S7-*Il1r1*^*tm1Imx*^/J mice (IL-1R KO) were infected with Theiler’s murine encephalomyelitis virus (1 x 10^6^ PFU). Differences in the development of demyelinating disease and changes in the histopathology were compared. Viral persistence, cytokine production, and immune responses in the CNS of infected mice were analyzed using quantitative PCR, ELISA, and flow cytometry.

**Results:**

Administration of IL-1β, thereby rending resistant B6 mice susceptible to TMEV-induced demyelinating disease, induced a high level of Th17 response. Interestingly, infection of TMEV into IL-1R-deficient resistant C57BL/6 (B6) mice also induced TMEV-induced demyelinating disease. High viral persistence was found in the late stage of viral infection in IL-1R-deficient mice, although there were few differences in the initial anti-viral immune responses and viral persistent levels between the WT B6 and IL-1R-deficiecent mice. The initial type I IFN responses and the expression of PDL-1 and Tim-3 were higher in the CNS of TMEV-infected IL-1R-deficient mice, leading to deficiencies in T cell function that permit viral persistence.

**Conclusions:**

These results suggest that the presence of high IL-1 level exerts the pathogenic role by elevating pathogenic Th17 responses, whereas the lack of IL-1 signals promotes viral persistence in the spinal cord due to insufficient T cell activation by elevating the production of inhibitory cytokines and regulatory molecules. Therefore, the balance of IL-1 signaling appears to be extremely important for the protection from TMEV-induced demyelinating disease, and either too much or too little signaling promotes the development of disease.

## Introduction

Toll-like receptors (TLRs) and interleukin-1 receptors (IL-1Rs) are involved in the production of various cytokines that are associated with the innate immune response against many different infectious agents. TLRs and IL-1Rs share many structural similarities and utilize common downstream adaptive molecules after activation by their ligands. In general, these innate immune responses induced by TLRs and IL-1Rs are known to play a protective role against various microbes [1]. However, several recent studies have indicated that these signals may also play a pathogenic role in viral infections [2-4]. In addition to TLRs, IL-1Rs are also considered to be important innate receptors because IL-1β, in particular, is a prominent cytokine that appears in the early stage of microbial infections [3]. The IL-1R family contains six receptors, including IL-1RI, which recognizes the principal inflammatory cytokine IL-1β and the less inflammatory cytokine IL-1α [1,5]. IL-1β is generated from the cleavage of pro-IL-1β by caspase-1 in inflammasomes after infections, and the downstream signaling cascade of the IL-1β-IL-1R interaction leads to the induction of various proinflammatory cytokines and the activation of lymphocytes [6]. IL-1β-deficient mice show broad host susceptibility to various infections [7,8]. Moreover, IL-1RI-deficient mice are susceptible to certain pathogens, including *Listeria monocytogenes*[1]. Therefore, these responses to IL-1β are apparently critical for protection from many types of viruses and microbes. However, the level of IL-1β has also been linked to many different inflammatory autoimmune diseases, including diabetes, lupus, arthritis, and multiple sclerosis (MS) [1,4].

Theiler’s murine encephalomyelitis virus (TMEV) is a positive-stranded RNA virus in the *Picornaviridae* family [9]. TMEV establishes a persistent CNS infection in susceptible mouse strains that results in the development of chronic demyelinating disease, and the system has been studied as a relevant viral model for human multiple sclerosis [10-12]. Cells infected with TMEV produce various proinflammatory cytokines, including type I IFNs, IL-6 and IL-1β [13]. TLR3 and TLR2 are involved in the production of these cytokines following infection with TMEV [14,15]. In addition, melanoma differentiation-associated gene 5 and dsRNA-activated protein kinase R are known to contribute to the production of proinflammatory cytokines [14,16]. These pathways also induce activation of caspase-1, leading to the generation of IL-1β and IL-1α, which contribute to further cytokine production, such as IL-6 promoting the development of pathogenic Th17 cells. Because IL-1β signals are associated with both host protection from viral infections and pathogenesis of inflammatory immune-mediated diseases, we here investigated the role of IL-1β-mediated signals in the development of TMEV-induced demyelinating disease.

We have previously reported that Th17 cells preferentially develop in an IL-6-dependent manner after TMEV infection, and that Th17 cells promote persistent viral infection and induce the pathogenesis of chronic demyelinating disease [17]. In addition, our earlier studies indicated that administration of either lipopolysaccharide (LPS) or IL-1β, thereby inducing high levels of IL-6 production, into resistant C57BL/6 (B6) mice renders the mice susceptible to the development of TMEV-induced demyelinating disease [18]. These results suggest that an excessive level of IL-1β is harmful to TMEV-induced demyelinating disease by generating high levels of pathogenic Th17 cells [19]. In this study, we confirmed the role of excessive IL-1β in the generation of a high level of Th17 cells in resistant B6 mice, supporting the pathogenic mechanisms of IL-1β. Furthermore, we have also utilized IL-1R-deficient mice to investigate the role of IL-1β-mediated signaling in the development of TMEV-induced demyelinating disease. Our results indicate that the lack of IL-1 signaling in resistant B6 mice also induced TMEV-induced demyelinating disease. The initial deficiencies in T cell function, including cytokine production and high viral persistence in the late stage of viral infection, were found in IL-1R-deficient mice. Therefore, the presence of an excessive amount of IL-1 plays a pathogenic role by elevating pathogenic Th17 responses, whereas the lack of IL-1 signals promotes viral persistence in the spinal cord, leading to chronic immune-mediated inflammatory disease.

## Materials and methods

### Animals

Female C57BL/6 mice were purchased from the Charles River Laboratories (Charles River, MA, USA) through the National Cancer Institute (Frederick, MD). Female B6.129S7-*Il1r1*^*tm1Imx*^/J mice (IL-1R knockout (KO)) were purchased from Jackson Laboratories (Bar Harbor, ME, USA). These mice were housed in the Animal Care Facility of Northwestern University. Experimental procedures that were approved by the Animal Care and Use Committee (ACUC) of Northwestern University in accordance with NIH animal care guidelines were used in this study.

### Synthetic peptides and antibodies

All peptides used were purchased from GeneMed (GeneMed Synthesis Inc, CA, USA) and used as described previously [20]. All antibodies used were purchased from BD Pharmingen (San Diego, CA, USA).

### Virus

The BeAn strain of TMEV was generated, propagated, and titered in BHK-21 cells grown in Dulbecco’s modified Eagle medium supplemented with 7.5% donor calf serum as previously described [21]. Viral titer was determined by plaque assays on BHK cell monolayers.

### Viral infection of mice and assessment of clinical signs

For intracerebral (i.c.) infection, 30 μl virus solution, containing 1×10^6^ pfu, was injected into the right cerebral hemisphere of 6 to 8 week-old mice (n = 10 per group) anesthetized with isoflurane. Clinical symptoms of disease were assessed weekly on the following grading scale: grade 0 = no clinical signs; grade 1 = mild waddling gait; grade 2 = severe waddling gait; grade 3 = moderate hind limb paralysis; grade 4 = severe hind limb paralysis and loss of righting reflex.

### Reverse-transcriptase PCR and real-time PCR

Total cellular RNA from the brain and spinal cord of infected SJL/J mice was isolated using Trizol® Reagent (Invitrogen, CA, USA). First-strand cDNA was synthesized from 1 μg total RNA utilizing SuperScript® III First-Strand Synthesis Supermix or M-MLV (Invitrogen). The cDNAs were amplified with specific primer sets using the SYBR Green Supermix (Bio-Rad) on an iCycler (Bio-Rad). Primers for control GAPDH and cytokine genes were purchased from Integrated DNA Technologies. GAPDH: forward primer, AACTTTGGCATTGTGGAAGG and reverse primer, ACACATTGGGGGTAGGAACA; VP-1: TGACTAAGCAGGACTA-TGCCTTCC and CAACGAGCCACATATGCGGATTAC; IFN-α: (5’-ACCTCCTCT-GACCCAGGAAG-3’ and 5’-GGCTCTCCAGACTTCTGCTC-3’); IFN-β: CCCTAT-GGAGATGACGGAGA and CTGTCTGCTGGTGGAGTTCA; CXCL10: (5’-AAGT-GCTGCCGTCATTTTCT-3’ and 5’-GTGGCAATGATCTCAACACG-3’); IL-10: GCCAAGCCTTATCGGAAATGATCC and AGACACCTTGGTCTTGGAGCTT; IFN-γ: ACTGGCAAAAGGATGGTGAC and TGA GCTCATTGAATGCTTGG; IL-17A: CTCCAGAAGGCCCTCAGACTAC and AGCTTTCCCTCCGCATTGACACAG; IL-6: AGTTGCCTTCTTGGGACTGA and TCCACGATTTCCCAGAGAAC; TNF-α: GGTCACTGTCCCAGCATCTT and CTGTGAAGGGAATGGGTGTT.

### Isolation of CNS-infiltrating mononuclear cells (MNCs)

Mice were perfused with sterile Hank’s balanced salt solution (HBSS), and excised brains and spinal cords of 3 mice per group were homogenized. CNS-infiltrating MNCs were then enriched in the one third bottom fraction of a continuous Percoll (Pharmacia, Piscataway, NJ, USA) gradient after centrifugation for 30 minutes at 27,000 *g* as described previously [22].

### Flow cytometry

CNS-infiltrating lymphocytes were isolated, and Fc receptors were blocked using 100 Î¼L of 2.4G2 hybridoma (ATCC) supernatant by incubating at 4°C for 30 minutes. Cells were stained with anti-CD8 (clone 53–6.7), anti-CD4 (clone GK1.5), anti-CD11b (clone M1/70), anti-NK1.1 (clone PK136), anti-GR-1 (clone RB6-8C5) and anti-CD45 (clone 30-F11) antibodies. All antibodies used for flow cytometry were purchased from BD Pharmingen (San Diego, CA). Cells were analyzed using a Becton Dickinson LSRII flow cytometer.

### Intracellular staining of cytokine production

Freshly isolated CNS-infiltrating MNCs from three mice per group were cultured in 96-well round-bottom plates in the presence of the relevant or control peptide as previously described [23]. Allophycocyanin-conjugated anti-CD8 (clone Ly2) or anti-CD4 (clone L3T4) antibodies and a PE-labeled rat monoclonal anti-IFN-γ (XMG1.2) antibody were used for intracellular cytokine staining. Cells were analyzed on a Becton Dickinson FACS Calibur or LSRII cytometer. Live cells were gated based on light scattering properties.

### T cell proliferation assay

Splenocytes from TMEV-infected mice, CD4^+^ T cells from spleens of OTII mice stimulated with specific epitope peptide, or *in vitro* TMEV-infected peritoneal macrophages in the presence of 2 μM ovalbumin (OVA)-specific peptides or 100 μg OVA protein were used. Cultures were incubated in 96-well flat-bottomed microtiter plates for 72 h and then pulsed with 1.0 μCi [^3^H]TdR and harvested 18 h later. [^3^H]TdR uptake by the cells was determined in triplicate using a scintillation counter and expressed as net counts per minute (Δcpm) ± standard error of the mean (SEM) after subtraction of the background count of cultures with PBS instead of stimulators.

### Histopathological analyses

At 70 days post-infection, mice were anesthetized and perfused via intracardiac puncture with 50 mL of PBS. Brain and spinal cords from IL-1R KO and B6 control mice were dissected and fixed in 4% formalin in PBS for 24 h. Anterior cortex (bregma: 3.0 to 2.0 mm), subventricular zone (bregma: 1.7 to 0.48), hippocampus (bregma: -1.0 to −2.5 mm), and cerebellum (bregma: -5.6 to −7.0 mm) were investigated. In addition, cervical, thoracic, and lumbar regions of the spinal cord were also examined. The tissues were embedded in paraffin for sectioning and staining. Paraffin-processed brains and spinal cords were sectioned at 6 μm. Adjacent sets of three sections from each animal were deparaffinized, rehydrated, and evaluated by H & E staining for inflammatory infiltrates, Luxol Fast Blue (LFB) staining for axonal demyelination, and Bielschowsky silver staining for axon loss. Slides were examined using a Leica DMR light microscope, and images were captured using an AxioCam MRc camera and AxioVision imaging software. The inflammatory infiltrates were evaluated by the presence or absence of the monocytes/lymphocytes based on the H & E staining and immunofluorescent staining of CD45^+^ cells. Histologic white matter demyelination was graded as: 1) normal myelination, 2) mild or minor demyelination (> 50% myelin staining preserved), or 3) moderate to severe demyelination (< 50% myelin staining preserved).

### ELISA

Cytokine levels produced by splenocytes from TMEV-infected mice or CD4^+^ T cells from spleens of OTII mice were determined after stimulation with specific epitope peptides (2 μM each), or *in vitro* TMEV-infected peritoneal macrophages in the presence of OVA-specific peptide (2 μM) for 72 h, respectively. IFN-γ (OPTEIA kit; BD Pharmingen), IL-17 (R&D Systems, Minneapolis, MN, USA) levels were assessed. Plates were read using a Spectra MAX 190 microplate reader (Molecular Devices, Sunnyvale, CA, USA) at a 450 nm wavelength.

### Statistical analysis

Data were presented as mean ± SD of either two to three independent experiments or one representative of at least three separate experiments. The significance of differences in the mean values was determined by Student’s *t*-test. Clinical scores were analyzed by Mann–Whitney *U*-test. *P* values < 0.05 were considered statistically significant.

## Results

### Administration of IL-1β promotes a Th17 response to TMEV to exacerbate the pathogenicity of demyelinating disease

**Figure 1:**
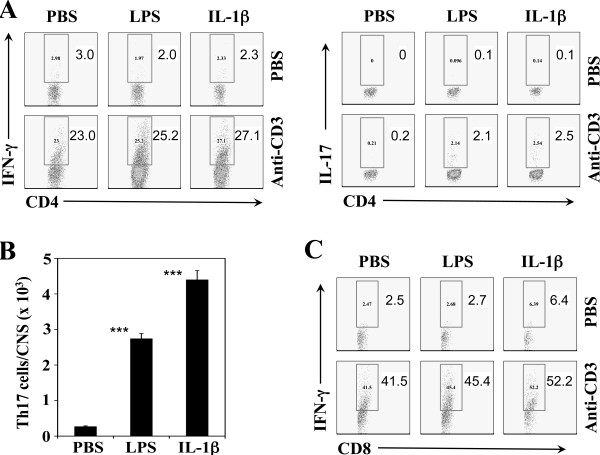
**Effects of IL-1β administration on Th17 and Th1 responses in TMEV-infected B6 mice.** (**A**) Levels of IFN-γ- and IL-17-producing CD4^+^ T cells in B6 mice intraperitoneally treated with PBS, lipopolysaccharide (LPS), or IL-1β during the early stage (−1 and 3 days post-infection (dpi)) of Theiler’s murine encephalitis virus (TMEV) infection (three mice per group) were analyzed using flow cytometry of the pooled central nervous system (CNS) cells at 8 dpi after stimulation with either PBS or anti-CD3/CD28 antibodies. (**B**) The overall numbers of IL-17-producing cells in the CNS of the above treated B6 mice (three mice per group) were shown. (**C**) Levels of IFN-γ-producing CD8^+^ T cells in the CNS of these groups of mice were also similarly analyzed. ***P < 0.001.

We have previously demonstrated that administration of LPS or IL-1β causes resistant C57BL/6 mice to develop demyelinating disease [18]. It has recently been shown that LPS treatment promotes this pathogenesis by elevating the induction of the pathogenic Th17 response [17]. However, it is unknown how IL-1β promotes this pathogenesis. To understand the mechanism, we compared the levels of Th1 and Th17 in the CNS of B6 mice treated with either LPS or IL-1β, along with control mice treated with PBS, following infection with TMEV at 8 days post-infection (Figure 1). The results clearly indicated that the levels of IL-17A-producing Th17 cells in mice treated with either LPS or IL-1β were significantly elevated compared to PBS-treated control mice (Figure 1A and B). In contrast, the levels of IFN-γ-producing Th1 cells were not different. It is interesting to note that IL-1β-treated mice exceeded the Th17 level of LPS-treated mice. However, the levels of IL-17-producing CD8^+^ T cells were minimal (not shown), and the levels of IFN-γ-producing CD8^+^ T cells were also similar among the groups (Figure 1C). These results strongly suggest that IL-1β can promote the pathogenesis of TMEV-induced demyelinating disease by enhancing the induction of pathogenic Th17 cells rather than altering the Th1 response.

### IL-1R KO mice are susceptible to TMEV-induced demyelinating disease and display high cellular infiltration to the CNS

**Figure 2:**
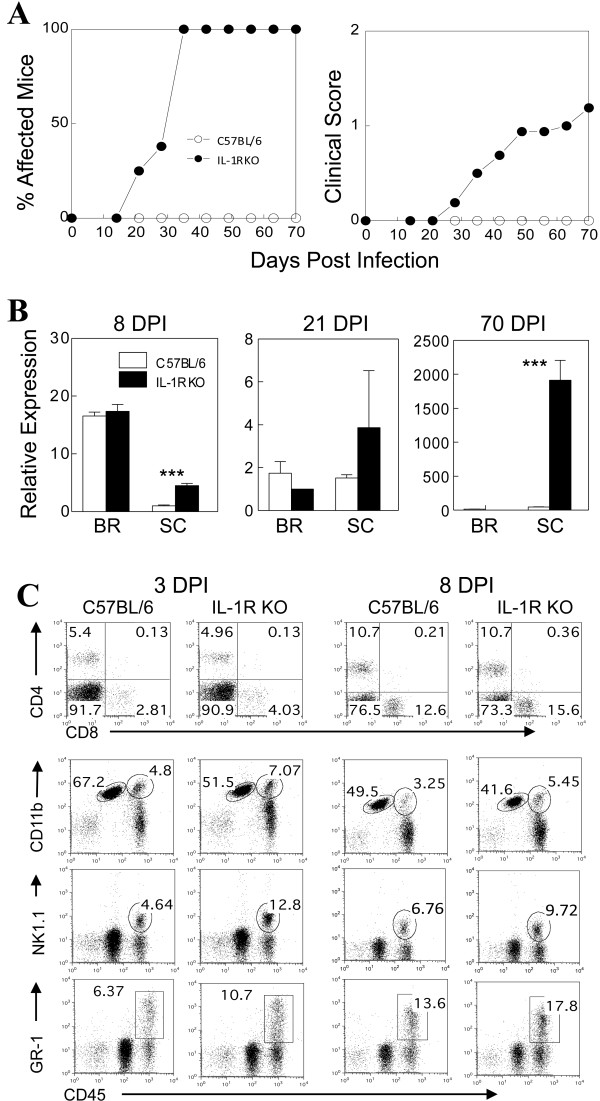
**The course of TMEV-induced demyelinating disease development, viral persistence levels and CNS-infiltrating mononuclear cells in TMEV-infected in B6 and IL-1R KO mice.** (**A**) Frequency and severity of demyelinating disease in B6 (n = 10) and IL-1R knockout (KO) (n = 10) mice were monitored for 70 days after Theiler’s murine encephalitis virus (TMEV) infection. (**B**) Viral persistence levels in the pooled brains (BR) and spinal cords (SC) of infected mice (three mice per group) at 8, 21 and 70 days post-infection (dpi) were determined by quantitative PCR. Data are expressed by fold induction after normalization to the glyceraldehyde-3-phosphate dehydrogenase (GAPDH) mRNA levels. The values are expressed as the means ± SD of triplicate experiments. Statistically significant differences are indicated with asterisks;*P < 0.05, **P < 0.01, ***P < 0.001. (**C**) Levels of T cells (CD4^+^ and CD8^+^), macrophages (CD11b^+^CD45^high^), microglia (CD11b^+^CD45^int^), NK cells (NK1.1^+^CD45^+^) and granulocytes (Ly6G/6C^+^CD45^+^) were assessed using flow cytometry in central nervous system (CNS)-infiltrating mononuclear cells from TMEV-infected C57BL/6 and IL-1R KO mice at 3 and 8 dpi. Numbers in FACS plots represent percentages in the CNS. Data are representative of three experiments using three mice per group.

Although administration of IL-1β promotes the pathogenesis of TMEV-induced demyelinating disease, the IL-1β produced via NLRP3 proteosome activation upon viral infection is considered to be a protective mechanism against microbial infections by promoting the apoptosis of infected cells [24]. To further investigate the potential role of IL-1β-mediated signaling in the development of TMEV-induced demyelinating disease, we compared the development of TMEV-induced demyelinating disease in IL-1R KO mice with a B6 background and control B6 mice (Figure 2A). Every IL-1R KO mouse developed demyelinating disease while none of the control B6 mice showed clinical signs at 35 days post-infection (dpi). The results clearly indicated that B6 mice with the deficiency in IL-1 signaling became susceptible to the TMEV-induced disease. This is somewhat unexpected because our previous study indicated that administration of IL-1β to B6 mice renders the mice susceptible to the disease, suggesting a pathogenic role for IL-1β in disease development.

To correlate the disease susceptibility of IL-1R KO mice with viral persistence in the CNS, the relative viral message levels in the CNS of wild type (WT) B6 and IL-1R KO mice were compared at days 8, 21 and 70 post-infection with TMEV (Figure 2B). The results showed that the level of viral load in the spinal cord, but not in the brain, is consistently higher in IL-1R KO mice compared to control B6 mice. These results strongly suggest that IL-1 signaling plays an important role in controlling viral persistence in the spinal cord during the course of TMEV infection. However, it was previously shown that the viral load level alone is not sufficient for the pathogenesis of TMEV-induced demyelinating disease [25]. Thus, we further assessed the levels of cellular infiltration to the CNS of these mice during the early stages (3 and 8 dpi) of viral infection (Figure 2C). The results indicated that infiltration into the CNS of granulocytes, NK cells, macrophages and CD8^+^ T cells but not CD4^+^ T cells was elevated, particularly at the early stage of viral infection. These results collectively suggest that high viral loads and cellular infiltration into the CNS in resistant B6 mice in the absence of IL-1 signaling leads to the elevated development of TMEV-induced demyelinating disease.

### IL-1R KO mice show widely spread mild demyelinating lesions accompanied by patchy axon damage

**Figure 3:**
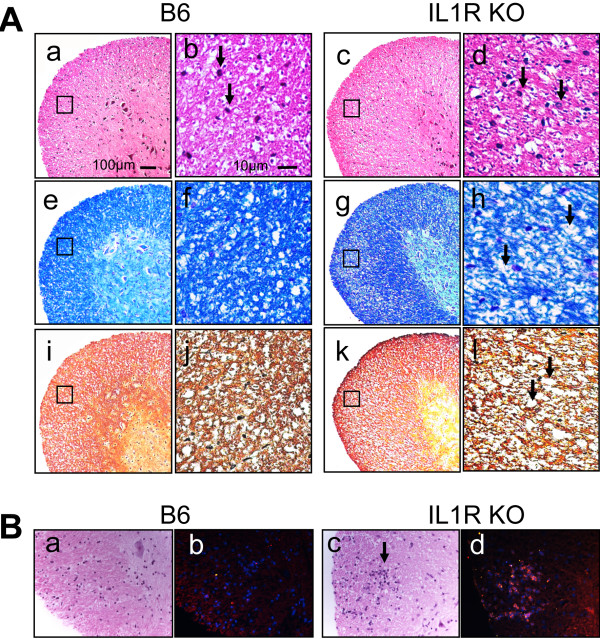
**Histopathology of the spinal cord in IL-1R KO and wild-type mice.** (**A**) H & E staining of the spinal cord showed infiltration of inflammatory cells in knockout (KO) mice (c, d) and little infiltration in wild-type (WT) mice (a, b). Luxol Fast Blue (LFB) staining of adjacent sections showed irregular vacuolation and minor demyelination in the white matter of KO mice (g, h) but no loss of myelin in WT mice (e, f). Bielschowsky silver staining of the same area shows the presence of irregular vacuolation and minor axonal loss in KO mice (k, l) but not in WT mice (i, j). Magnification, ×10 and ×40. Black arrows indicate regions of lymphocyte infiltrates, demyelination, or axon loss; thin black squares indicate the areas from the lumbar spinal cord region, which are shown in high magnification (b, f, and j for B6 mice and d, h, and l for IL-1R KO mice). (**B**) H & E staining of spinal cords of control (a) and IL-1R KO mice (c) are shown. The adjacent sections (b and d, respectively) were stained with anti-CD45 antibody (red) for infiltrating cells and counterstained with 4',6-diamidino-2-phenylindole (DAPI) (blue) for nuclei.

At 70 days post-infection, the histopathology of TMEV-infected IL-1R KO and B6 mice was compared to correlate the disease development with the histopathology of the CNS (Figure 3). Series of histopathological examinations of the spinal cords from both KO and WT mice were conducted after H & E, LFB, and Bielschowsky silver staining. The H & E staining was used for evaluating the evidence of active inflammation and lymphocyte infiltration. LFB specifically stains axonal myelin sheath, and this was used to evaluate the axonal demyelination. Bielschowsky silver staining stains axons dark brown and was used to evaluate axonal integrity. Lymphocyte infiltration, minor demyelination and axon loss were detected in the CNS, including the brain and spinal cord, in IL-1R KO mice but not in WT B6 mice. Compared to control B6 mice (Figure 3A a-b), IL-1R KO mice (Figure 3A c-d) showed more lymphocyte infiltration in the white matter of the lumbar spinal cord when examined by H & E staining. LFB staining of the adjacent sections showed irregular vacuoles and demyelination in the white matter of the spinal cord in IL-1R KO mice (Figure 3A g-h) and in brain regions including the cerebellum and medulla (not shown). In contrast, myelin that appeared normal and little histopathological change were observed in the control B6 mice (Figure 3A e-f). Bielschowsky silver staining of the adjacent sections also showed irregular vacuolation and mild axon loss in the demyelinated regions of the spinal cord from IL-1R KO mice (Figure 3A k-l) but not in the sections from the WT control mice (Figure 3A i-j). To further compare the cellular infiltration levels in the CNS of these mice, we examined the levels of CD45^+^ cells in the CNS which largely represents infiltrating cells (Figure 3B). Our results clearly displayed that the level of CD45^+^ cells (Figure 3B d), many of which overlap with H & E staining Figure 3B c), was higher in the CNS of IL-1R KO mice compared to that of the control B6 mice (Figure 3B a-b).

### Cytokine gene expression is transentily higher in the CNS of IL-1R KO mice during early viral infection

**Figure 4:**
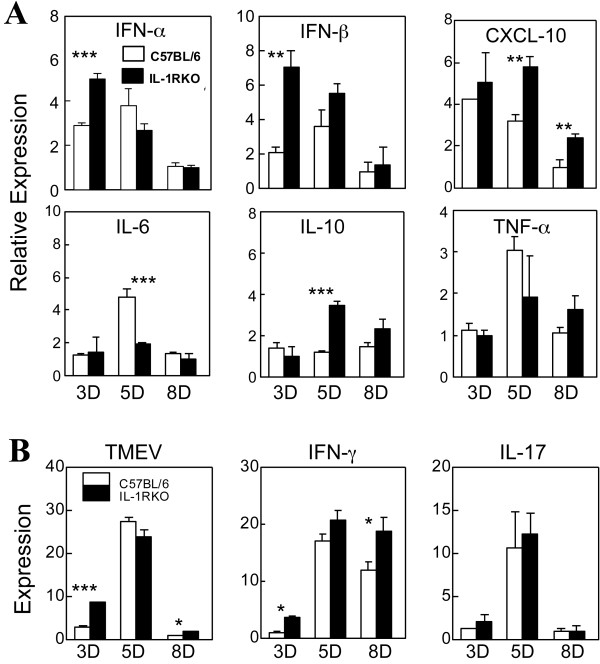
**Expression level of cytokine genes in the CNS of TMEV infected B6 and IL-1R knockout (KO) mice at 3, 5 and 8 days post-infection (dpi).** The relative expression levels of the indicated mRNAs in the central nervous system (CNS) of Theiler’s murine encephalitis virus (TMEV)-infected C57BL/6 and IL-1R KO mice at 3, 5 and 8 dpi were assessed by real-time PCR. Data are expressed by fold induction after normalization to the glyceraldehyde-3-phosphate dehydrogenase (GAPDH) mRNA levels. The values are expressed as the means ± SD of triplicates. Statistically significant differences are indicated with asterisks; *P < 0.05, **P < 0.01, ***P < 0.001).

To understand the susceptibility to TMEV-induced demyelinating disease in IL-1R KO mice, we analyzed various cytokine message levels expressed in the CNS of virus-infected control and IL-1R KO mice during the early stages (3, 5, and 8 dpi) of viral infection using real-time PCR (Figure 4). The levels of IFN-α and IFN-β gene expression in IL-1R KO mice were significantly higher than those in B6 mice at 3 dpi, although the levels became similar at 5 and 8 dpi. The expression levels of CXCL-10 that were associated with T cell infiltration and IL-10 that was associated with viral persistence were higher at 5 dpi, and this trend was maintained at 8 dpi. However, the expression level of IL-6 in IL-1R KO mice was transiently lower at 5 dpi, while no differences in TNF-α expression were noted. Similarly, the production of a pathogenic T cell cytokine, IL-17 was largely unchanged. However, viral RNA and the production of IFN-γ were transiently higher at 3 and 8 dpi. These results suggest that the lack of IL-1 signaling differentially affects viral replication and the expression of various innate and immune cytokines depending on the stage of TMEV infection.

### Anti-viral CD4^+^ T cell responses in the CNS, of virus-infected IL-1R KO mice are lower during the early stage of infection

**Figure 5:**
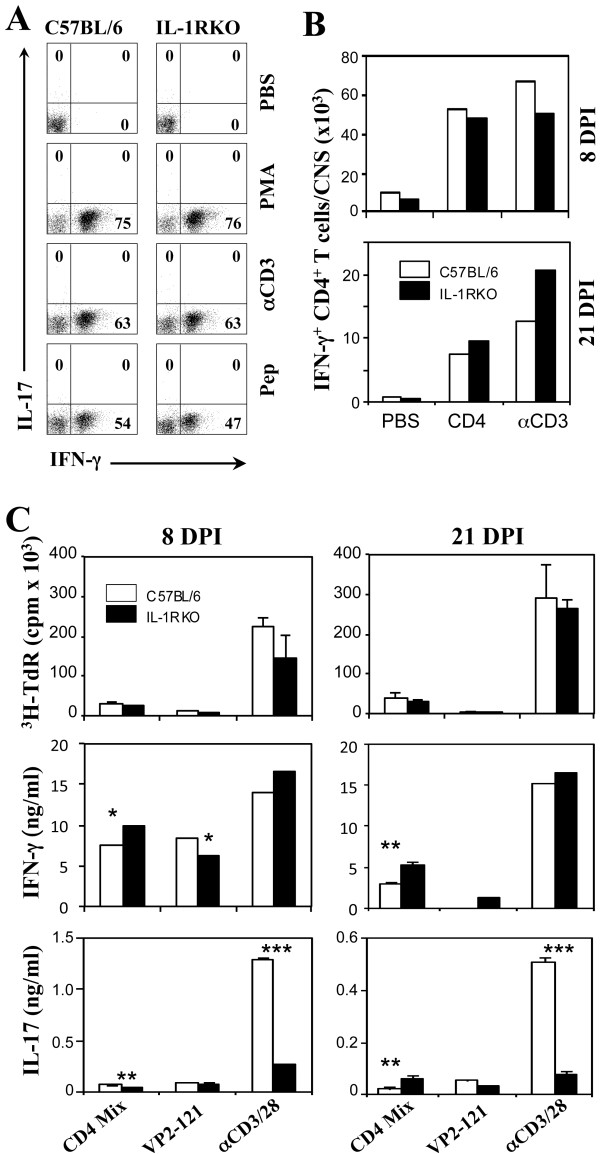
**Virus-specific CD4**^**+**^**T cell responses in the CNS and the periphery of TMEV-infected B6 and IL-1R knockout (KO) mice.** (**A**) Levels of Th1 and Th17 cells in the central nervous system (CNS) of virus-infected B6 and IL-1R KO mice were assessed using flow cytometry at 8 days post-infection (dpi) after stimulation with PBS, PMA/ionomycin, anti-CD3/CD28 antibodies, or viral epitope peptides (Pep: mixture of 2 μM VP2_203-220_ and 2 μM VP4_25-38_). The flow cytometric plots show gated CD4^+^ T cells. (**B**) The numbers of CNS-infiltrating IFN-γ-producing CD4^+^ T cells reactive to either viral epitope peptides (CD4: equal mixture of VP2_203-220_ and VP4_25-38_) or anti-CD3/CD28 antibodies in virus-infected B6 and IL-1R KO mice (three mice each) at 8 and 21 dpi. A representative result from two to three similar experiments is shown here. (**C**) Levels of proliferation and cytokine production by splenic T cells in response to viral epitopes by CD4^+^ T cells (CD4 Mix: equal mixture of VP2_203-220_ and VP4_25-38_), CD8^+^ T cells (VP2-_121–130_), or both CD4^+^ and CD8^+^ T cells (anti-CD3/CD28 antibodies) were assessed at 8 and 21 dpi. Values are expressed as the mean of triplicate samples (mean ± SD) from a representative of three experiments. *P < 0.05, **P < 0.01, ***P < 0.001.

To compare CD4^+^ T cell responses specific to viral determinants in the CNS of IL-1R KO and WT mice, infiltration levels of CD4^+^ T cells specific to the predominant CD4^+^ T cell epitopes were assessed (Figure 5A and B). The levels of IFN-γ-producing CD4^+^ T cells in response to pan-T cell stimulation (either PMA plus ionomycin or anti-CD3 and anti-CD28 antibodies) were similar between IL-1R KO and control WT B6 mice. However, such CD4^+^ T cell responses to viral epitopes were proportionally lower in the CNS of virus-infected IL-1R KO mice compared to B6 mice, although the overall levels in the CNS were similar. This discrepancy may be due to the high levels of CXCL-10 expression (Figure 4), which promotes infiltration of T cells, in the CNS of IL-1R KO mice. The levels of IL-17-producing CD4^+^ T cells in either TMEV-infected IL-1R KO or B6 mice were undetectable. To further determine whether the pattern of CD4^+^ T cell responses is unique in the CNS of virus-infected mice, we also assessed the T cell responses in the periphery of TMEV-infected IL-1R KO and control B6 mice at 8 and 21 dpi (Figure 5C). Again, the levels of T cell proliferation and the production of key T cell cytokines (IFN-γ and IL-17) against viral epitopes (for both CD4^+^ and CD8^+^ T cells) were not drasticaly different between the splenic T cells from IL-1R KO and control B6 mice. Despite the similar low levels of IL-17 production in response to viral epitopes, the IL-17 level was significantly lower in IL-1R KO mice compared to WT B6 mice after robust stimulation with anti-CD3/CD28 antibodies. These results are consistent with the role of IL-1 signaling in promoting IL-17 production [26,27].

### Levels of TMEV-specific CD8^+^ T cell responses in the CNS are comparable between IL-1R KO and WT B6 mice

**Figure 6:**
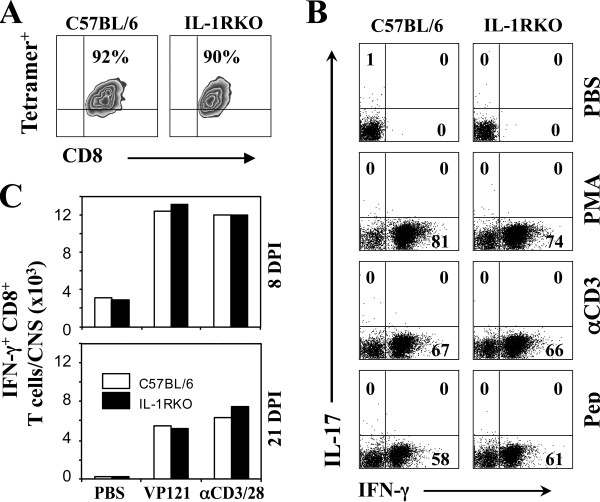
**Levels of virus-specific CD8**^**+**^**T cell responses in the CNS of virus-infected B6 and IL-1R KO mice.** (**A**) Levels of H-2D^b^-VP2-_121–130_-tetramer reactive CD8^+^ T cells in the central nervous system (CNS) of B6 and IL-1R knockout (KO) mice at 8 days post-infection (dpi). (**B**) Proportions of CNS-infiltrating CD8^+^ T cells reactive to viral epitopes, anti-CD3/CD28 antibodies and PMA/ionomycin were assessed using flow cytometry following intracellular cytokine staining at 8 dpi. (**C**) The overall numbers of virus-specific and anti-CD3/CD28 reactive CD8^+^ T cells in the CNS of virus-infected B6 and IL-1R KO mice are shown at 8 and 21 dpi. A representative result from two to three similar experiments is shown here.

To further determine whether the susceptibility of IL-1R KO mice to TMEV-induced demyelinating disease is associated with a compromised anti-viral CD8^+^ T cell response, we also analyzed the T cell responses in the CNS of TMEV-infected IL-1R KO and control B6 mice (Figure 6). Virus-specific CD8^+^ T cells reactive to the predominant epitope (VP2_121-130_), determined using the VP2_121_-H-2D^b^ tetramer, indicated that the proportions of virus-specific CD8^+^ T cells in the CNS of virus-infected WT B6 and IL-1R KO mice are similar (Figure 6A). To further determine whether the functions of the virus-reactive CD8^+^ T cells are different, we assessed the abilities of the cells to produce IFN-γ in response to specific and non-specific stimulations (Figure 6B). The results clearly indicated that their ability to produce IFN-γ is also similar in both proportion (Figure 6B) and number in the CNS of TMEV-infected B6 and IL-1R KO mice (Figure 6C). These results strongly suggest that there are no significant differences in the CD8^+^ T cell responses to the viral determinants, unlike those of CD4^+^ T cell responses, in the CNS of virus-infected WT B6 and IL-1R KO mice.

### Cytokine production by Th cells stimulated with macrophages from IL-1R KO mice is reduced

**Figure 7:**
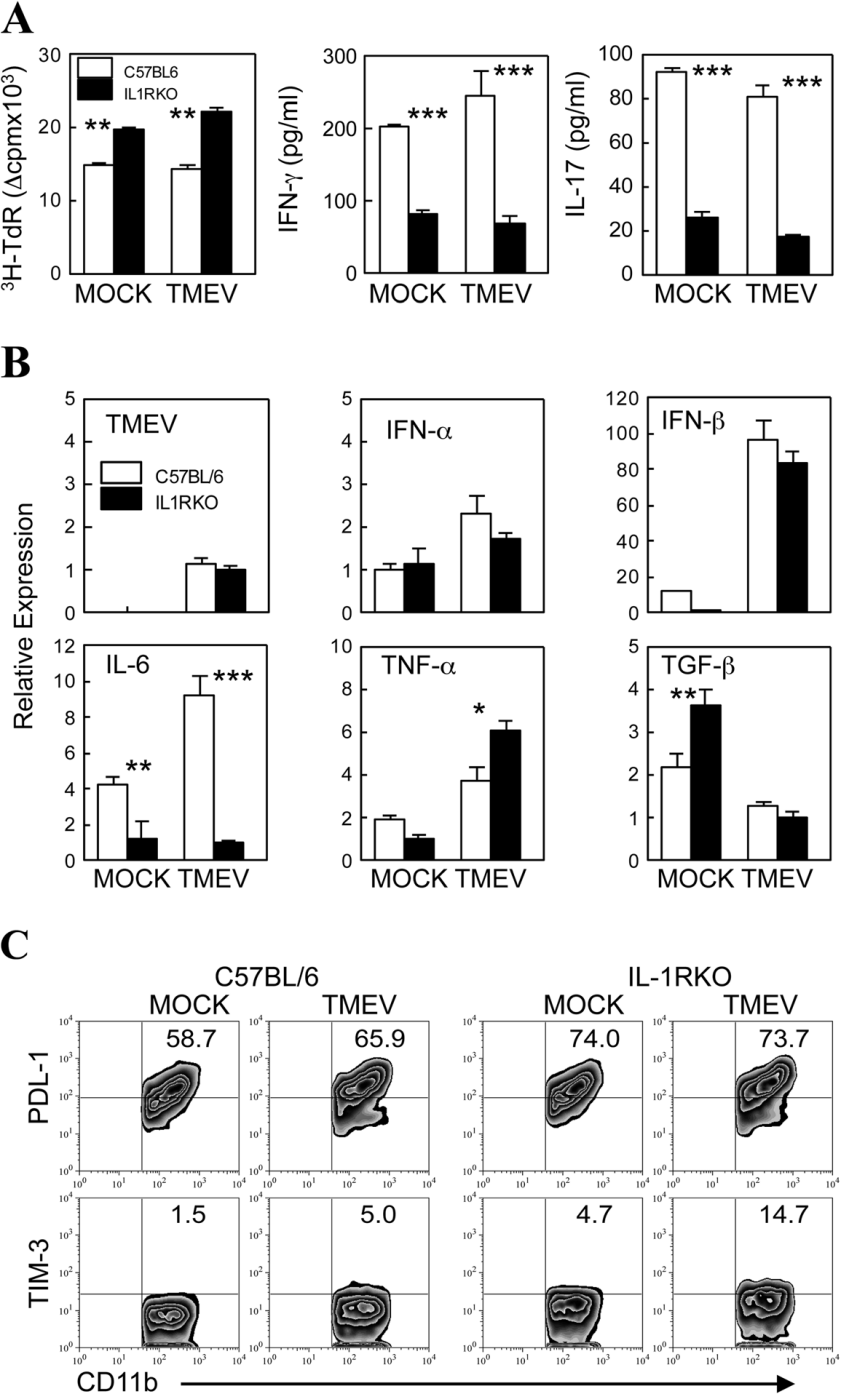
**Cytokine reduction of CD4**^**+**^**T cells with stimulation of IL-1R KO macrophages.** (**A**) Isolated CD4^+^ T cells (1 × 10^5^) from the spleen of OT-II mice were cultured with *in vitro* 10 MOI Theiler’s murine encephalitis virus (TMEV)-infected peritoneal macrophages (1 × 10^4^) from either C57BL/6 or IL-1R knockout (KO) mice for 3 days in the presence of 2 μM OVA epitope peptides. T cell proliferative responses were analyzed using [^3^H]TdR uptake, and cytokine production (IFN-γ and IL-17) of the cultures were analyzed using specific ELISAs. (**B**) Isolated CD4^+^ T cells (1 × 10^5^) from the spleen of B6 mice infected with TMEV at 8 days post-infection (dpi) were cultured with *in vitro* TMEV-infected (10 MOI for 24 h) peritoneal macrophages (1 × 10^4^) from either naïve C57BL/6 or IL-1R KO mice for 3 days in the presence of viral epitope peptides. Message levels of the indicated genes were then analyzed by real-time PCR. The glyceraldehyde-3-phosphate dehydrogenase (GAPDH) level was used as an internal control. The values are expressed as the means ± SD of triplicates. Statistically significant differences are indicated with asterisks; *P < 0.05, **P < 0.01, ***P < 0.001. Data shown are the representation of three independent experiments. (**C**) Peritoneal CD11b^+^ cells from naïve B6 and IL-1R KO mice infected with TMEV *in vitro* for 24 h were analyzed for the expression of PDL-1 and TIM-3 T cell inhibitory molecules. A representative flow cytometry plot of three similar results is shown here.

To compare the function of CD4^+^ T cells stimulated by macrophages from B6 and IL-1R KO mice, T cells from naïve OT-II mice, which carry T cell receptor (TCR) transgenes specific for OVA_323-339_, were stimulated with peritoneal macrophages infected *in vitro* for 24 h with TMEV (10 MOI) in the presence of OVA_323-339_ peptide (Figure 7A) or OVA protein (not shown). Viral infection did not significantly alter the levels of T cell stimulation by these macrophages. However, proliferation of CD4^+^ T cells in response to the cognate peptide or protein was higher when stimulated with IL-1R KO macrophages compared to the proliferation stimulated with B6 macrophages. In contrast, IFN-γ and IL-17 production by the T cells stimulated with IL-1R KO macrophages were significantly lower than the production stimulated with control B6 macrophages. These results indicated that antigen-presenting cells display altered T cell stimulating function in the absence of IL-1R-mediated signaling.

To further understand the potential mechanisms underlying the altered T cell stimulation by IL-1R KO macrophages, we examined the potential contribution of IL-1 signaling to the induction of cytokines in TMEV-infected macrophages (Figure 7B). After TMEV infection *in vitro* for 24 h, levels of viral RNA as well as IFN-α and IFN-β messages were similar between macrophages from WT B6 and IL-1R KO mice. However, the expression of the IL-6 message was extremely compromised in IL-1R KO macrophages compared to B6 macrophages. In contrast, the expression level of TNF-α message was more highly upregulated in IL-1R KO macrophages after TMEV infection. Interestingly however, the expression of TGF-β in uninfected IL-1R KO macrophages was higher than the expression in B6 macrophages but reduced to a similar level after viral infection. These results suggest that the cytokine production profile of macrophages and perhaps other antigen-presenting cells is altered in the absence of IL-1 signaling, which may affect the initial development and/or function of T cells following viral infection.

To further understand the underlying mechanisms associated with altered CD4^+^ T cell development in the absence of IL-1 signaling, the levels of co-stimulatory molecules and key inhibitory molecules were studied in a representative macrophage antigen-presenting cell (APC) population (Figure 7C). The levels of CD80, CD86 and CD40 in B6 and IL-1R1 KO mice were not significantly different (not shown). However, PDL-1 and Tim-3 were significantly elevated in the absence of IL-1 signaling. These molecules are known to negatively affect the function of T cells [28] and/or promote inflammatory responses through its expression by innate immune cells, such as microglia [29]. It was interesting to note that the expression of PDL-1 was upregulated upon viral infection in B6 macrophages, whereas the expression in IL-1R-deficient macrophages was constitutively upregulated even in the absence of viral infection. In contrast, the expression of Tim-3 was constitutively upregulated in IL-1R-deficient macrophages to the level (approximately 5%) of upregulation seen after viral infection in B6 macrophages, but it was further upregulated in IL-1R KO macrophages after TMEV infection. These results strongly suggest that these increases in the inhibitory molecules may participate in the altered T cell development and/or function.

## Discussion

TMEV-infection in susceptible strains of mice induces chronic demyelinating disease that is primarily mediated by CD4^+^ T cells [17,30,31]. However, epitope-specific CD4^+^ T cells can be protective or pathogenic depending on when activated T cells are available in conjunction with viral infection [23,32,33]. Interestingly, the level of IL-1β, induced following infection with TMEV, plays an important role in the pathogenesis of TMEV-induced demyelinating disease [18,34]. Previously, it has been shown that administration of IL-1 to mice exacerbates the development of experimental autoimmune encephalomyelitis (EAE), the pathogenic immune mechanisms of which are similar to those of TMEV-induced demyelinating disease [35-37]. In addition, IL-1 appears to directly activate astrocytes and microglia to exacerbate neurodegeneration in non-immune-mediated diseases [38]. Because IL-1β is induced via the innate immunity mediated by various TLRs and because the downstream IL-1 signals mediated via IL-1R also play an important role in the host defense [1,4], we have investigated the role of IL-1β signals in the development of TMEV-induced demyelinating disease by assessing the effects of IL-1β administration and using IL-1R-deficient mice.

We have previously demonstrated that administration of IL-1β into resistant B6 mice renders the resistant mice susceptible to TMEV-induced demyelinating disease [18]. The administration of IL-1β dramatically increased the level of IL-17 production in the CNS of the resistant mice, which do not produce a high level of Th17 cells following TMEV infection (Figure 1). This result is consistent with recent reports that IL-1β strongly promotes the development of IL-17-producing Th17 cells either directly or via the production of IL-6 [19,39]. The presence of high levels of IL-17A in mice infected with TMEV exerts a strong pathogenic role by inhibiting the apoptosis of virus-infected cells, blocking cytolytic CD8^+^ T cell function, and elevating cellular infiltration to the CNS [17]. Recently, it was also shown that the presence of FoxP3^+^ Treg cells that preferentially expand due to stimulation by IL-1β [40] is not beneficial for the development of TMEV-induced demyelinating disease; hence, these regulatory cells inhibit the protective anti-viral immune responses [41]. Therefore, administration of IL-1β, resulting in a higher level of IL-1β, appears to promote the pathogenesis of TMEV-induced demyelinating disease in resistant B6 mice by elevating pathogenic Th17 and Treg responses to TMEV antigens. In addition, it is known that IL-1 directly activates astrocytes and microglia in the CNS [42], which are associated with the pathogenesis of TMEV-induced demyelinating disease [13,43]. Furthermore, IL-1 mediates the loss of astroglial glutamate transport and drives motor neuron injury in the spinal cord during viral encephalomyelitis [44]. The expression of IL-1R1 is upregulated in glial cells following TMEV infection [45], and thus the elevated receptor expression is likely to exert the detrimental effects seen as a result of IL-1 signaling on neurodegeneration and/or pathogenic immune responses.

In the absence of IL-1R1-mediated signals, resulting from engagements with the predominant cytokine IL-1β and weak cytokine IL-1α, strongly resistant B6 mice become susceptible to the development of TMEV-induced disease (Figure 2). Viral loads in the spinal cord are higher in the absence of IL-1R signals, suggesting that the presence of IL-1 signaling plays an important role in controlling viral persistence during the course of TMEV infection. The high viral loads also accompanied higher cellular infiltration into the CNS. Histopathological examinations of the virus-infected IL-1R-deficient B6 mice confirmed the elevated lymphocyte infiltration, demyelination and axonal losses in the CNS compared to control B6 mice (Figure 3). These results are consistent with previous reports indicating that either IL-1β- or IL-1RI-deficient mice are susceptible to various infections [1,7,8,46]. These results collectively suggest that either an abnormally high level of IL-1β or the absence of IL-1-mediated signals lead to high viral loads and cellular infiltration to the CNS, resulting in the elevated development of TMEV-induced demyelinating disease. Therefore, a fine balance of IL-1β-mediated signaling appears to be important for protection from viral infections. It is also interesting to note that this viral model for MS is markedly different from the EAE model, which is not associated with microbial infections, in that a deficiency of IL-1R1 significantly reduces the development of demyelinating disease [37].

Despite many previous studies on the role of IL-1β signaling in viral infections, the underlying mechanisms of the signals involved in the protection from infection remain unclear. Previously, it has shown that IL-1-mediated signals augment T cell responses by increasing cellular infiltration, as well as upregulating cytokine production and co-stimulatory molecule expression in APCs [5,47,48]. However, our results showed that the cellular infiltration is elevated in IL-1R1 KO mice during the early stages of viral infection (Figure 2), although the anti-viral CD4^+^ T cell responses in the CNS of virus-infected IL-1R KO mice are lower without compromising either peripheral CD4^+^ T cell responses (Figure 5) or CNS CD8^+^ T cell responses (Figure 6). These results suggest that the APCs associated with CD4^+^ T cell responses in the CNS are primarily affected by the absence of IL-1-mediated signaling. Our previous studies strongly suggested that primarily the microglia and, to a certain extent, astrocytes, harbor viral loads and play important roles in the stimulation of the level and type of the CD4^+^ T cell response [43]. In addition, it is known that IL-1 signaling affects the function of these cell types [42]. Therefore, it is most likely that these cells play an important role in the development of anti-viral CD4^+^ T cell responses in the CNS during the early stage of viral infection. Because the cytokine production profile of APCs is altered in the absence of IL-1 signaling, perhaps due to the elevated expression of inhibitory molecules (Figure 7), similar mechanisms by CNS APCs may negatively affect the initial development and/or function of anti-viral T cells following viral infection. Regarding the underlying mechanisms, it is currently unclear how the deficiency in IL-1 signals enhances the expression of inhibitory molecules in APCs. However, we have observed that APCs from susceptible SJL mice expressed significantly higher levels of these molecules upon viral infection either *in vitro* or *in vivo* compared to cells from resistant B6 mice (data not shown), suggesting that the viral load may lead to the elevated expression. Therefore, it is most likely that the absence of IL-1 signals permits the initial elevation of viral load (Figure 4), and the higher viral load, in turn, leads to an eventual compromise in the efficiency of anti-viral T cell responses and functions. In contrast, the presence of excessive IL-1 signals preferentially triggers T cell responses that are unfavorable for the protection of the hosts from chronic viral persistence and the pathogenesis of demyelinating disease, as previously seen [17,19].

## Conclusions

IL-1 signaling plays a protective role against viral infections. However, we have previously demonstrated that administration of IL-1 promotes the pathogenesis of TMEV-induced demyelinating disease, similar to the autoimmune disease model (EAE) for MS. The IL-1-mediated pathogenesis of TMEV-induced demyelinating disease appears to reflect an elevated Th17 response in the presence of IL-1. However, IL-1R-deficient B6 mice also induced TMEV-induced demyelinating disease accompanied by high viral persistence and upregulated expression of T cell inhibitory molecules such as PDL-1 and Tim-3. These results suggest that the presence of high IL-1 level promotes the pathogenesis by elevating Th17 responses, whereas the absence of IL-1 signals permits viral persistence in the CNS due to insufficient T cell activation. Therefore, the balance of IL-1 signaling appears to be critical for the determination of protection vs. pathogenesis in the development of a virus-induced demyelinating disease.

## Abbreviations

APC: antigen-presenting cell; CNS: central nervous system; Dpi: days post-infection; EAE: experimental autoimmune encephalomyelitis; ELISA: enzyme-linked immunosorbent assay; GAPDH: glyceraldehyde-3-phosphate dehydrogenase; H & E: hematoxylin and eosin; IL-1R: interleukin-1 receptor; LFB: Luxol Fast Blue; LPS: lipopolysaccharide; MNC: mononuclear cell; MS: multiple sclerosis; OVA: ovalbumin; PBS: phosphate-buffered saline; PCR: polymerase chain reaction; PFU: plaque-forming unit; SEM: standard error of the mean; TLR: toll-like receptor; TMEV: Theiler’s murine encephalomyelitis virus; TMEV-IDD: TMEV-induced demyelinating disease.

## Competing interests

The authors declare that they have no competing interests.

## Authors’ contribution

BSK directed experiments, interpreted the results and wrote the manuscript. YHJ conducted immunological experiments and helped writing. LM conducted histological experiments and wrote the corresponding portions. HSK performed some molecular analyses. WH and HSP conducted the initial immunological experiments. CSK contributed for the interpretation of results and direction of the study. All authors read and approved the final manuscript.
